# Breastfeeding practices in Northeast China in 2008 and 2018: cross-sectional surveys to explore determinants over a decade

**DOI:** 10.1186/s13006-023-00562-4

**Published:** 2023-05-02

**Authors:** Qianling Zhou, Xing Lin Feng

**Affiliations:** 1 Department of Maternal and Child Health, School of Public Health, Peking University, Beijing, 100191 China; 2 Department of Health Policy and Management, School of Public Health, Peking University, No. 38, Xueyuan Road, Haidian District, Beijing, 100191 China

**Keywords:** Prevalence, Determinant, Breastfeeding, Early initiation of breastfeeding, Northeast China

## Abstract

**Background:**

This study was conducted to investigate the prevalence and determinants of breastfeeding in 2008 and 2018, respectively, in Northeast China, where health service efficiency is at the lowest national level and regional data on breastfeeding are lacking. The influence of early initiation of breastfeeding on later feeding practices was specifically explored.

**Methods:**

Data from the China National Health Service Survey in Jilin Province in 2008 (n = 490) and 2018 (n = 491) were analysed. Multistage stratified random cluster sampling procedures were used to recruit the participants. Data collection was conducted in the selected villages and communities in Jilin. Early initiation of breastfeeding was defined as the proportion of children born in the last 24 months who were put to the breast within one hour after birth in both the 2008 and 2018 surveys. Exclusive breastfeeding was defined as the proportion of infants 0–5 months of age who were fed exclusively with breast milk in the 2008 survey; while defined as the proportion of infants 6–60 months of age who had been fed exclusively with breast milk within the first six months of life in the 2018 survey.

**Results:**

The prevalence of early initiation of breastfeeding (27.6% in 2008 and 26.1% in 2018) and exclusive breastfeeding during the first six months (< 50%) were low in two surveys. Logistic regression revealed that exclusively breastfeeding at six months was positively associated with early initiation of breastfeeding (OR 2.65; 95% confidence interval (CI) 1.65, 4.26) and negatively associated with caesarean section (OR 0.65; 95% CI 0.43, 0.98) in 2018. Continued breastfeeding at one year and timely introduction of complementary foods were associated with maternal residence and place of delivery, respectively, in 2018. Early initiation of breastfeeding was associated with mode and place of delivery in 2018 but residence in 2008.

**Conclusion:**

Breastfeeding practices in Northeast China are far from optimal. The negative effect of caesarean section and positive effect of early initiation of breastfeeding on exclusive breastfeeding suggest that an institution-based approach should not be substituted by the community-based one in the formulation of breastfeeding strategies in China.

**Supplementary Information:**

The online version contains supplementary material available at 10.1186/s13006-023-00562-4.

## Background

Optimal breastfeeding affects children’s nutrition and health [1, 2]. The World Health Organization (WHO) and United Nations Children’s Fund (UNICEF) recommend early initiation of breastfeeding (i.e., providing breast milk to infants within one hour after birth), exclusive breastfeeding (i.e., feeding the child with breast milk only, but not anything else. However, the child is also to receive oral rehydration solution, drops, or vitamins / minerals / medicines syrups) during the first six months of life, introduction of complementary foods at six months, and continuing breastfeeding up to two years and beyond [3]. Until 2020, however, only 44% of infants on the globe were breastfed exclusively within six months, 48% were breastfed within the first hour after birth, 69% were breastfed at one year of age, and 69% of infants 6–8 months of age were introduced to solid, semi-solid or soft foods [4].

In China, breastfeeding practices are far from optimal [5]. For example, a population-based survey (n = 10,408) conducted in 12 provinces / municipalities in 2017–18 reported that less than 10% of children received early initiation of breastfeeding, and no more than 30% were exclusively breastfed within six months [6, 7]. Data from the Under-5 Child Nutrition and Health Surveillance System (U5CNHSS) demonstrated slightly higher coverage, with higher prevalence of exclusive breastfeeding (According to the WHO definition, “exclusive breastfeeding” was taken to mean feeding of infants 0–5 months old with breast milk but neither liquid foods nor solid / semi-solid / soft food during the previous 24 h) observed in the East (39.3%) than the West (25.7%) [8]; however, the rates still lagged behind the global average. The prevalence and determinants of breastfeeding vary widely across geographical regions in China. For example, the prevalence of exclusive breastfeeding was higher in the east than in the west region [8]. The national surveys mentioned above both reported negative associations between maternal education and exclusive breastfeeding [9, 10], while data from Nanning city otherwise reported a positive association [11]. To date, regional data on breastfeeding prevalence and determinants are available only within Southeast China (Nanning city) [11], while relevant information from the northeastern part is still lacking.

In China, public health strategies that promote optimal breastfeeding practices have changed over the past three decades. Enlighted by the WHO and UNICEF, the baby-friendly hospital initiative (BFHI) was nationally scaled up during the 1990 to 2000 s [12], with an expectation to promote early initiation of breastfeeding [13]. However, along with major health system reform since 2009, the government’s efforts have shifted to community health care [14]. Breastfeeding was thus promoted through free essential public health services via antenatal and postnatal care [15–17]. Partly limited by the late recognition of the importance of exclusive breastfeeding [2, 18, 19], evidence is lacking on whether early initiation of breastfeeding or antenatal / postnatal care could better promote optimal breastfeeding practices. To our knowledge, no study has been conducted to compare the determinants of breastfeeding in China against the context of the 2008–18 decade when such a strategy shift was witnessed. In addition, there existed regional heterogeneity in the effectiveness of medical and health resource allocation over China’s health system reform, and the Northeast was reported to have the lowest health service efficiency [20]. Research should thus be conducted in the northeastern region to identify breastfeeding determinants and formulate effective and sensitive strategies for breastfeeding promotion.

In this study, we explored the prevalence and determinants of breastfeeding in Northeast China in 2008 and 2018, respectively. Using data from the provincial survey conducted in each of these two years, we compared the varied determinants of breastfeeding practices, including exclusive breastfeeding at six months; continued breastfeeding at one year; longer duration of breastfeeding; and timely introduction of complementary foods; while contextualizing China’s recent decade of efforts that focus on community-based health promotion of breastfeeding.

## Methods

### Data source

Data from China’s National Health Services Surveys (NHSS) conducted in Jilin Province in 2008 and 2018 were analysed separately. The NHSS surveys in Jilin are an official data source of the Ministry of Health of China that monitors the trends in health needs and coverage. Jilin Province (27,120 km^2^) is situated in the central part of Northeast China and characterised by its developed processing and manufacturing industries. The economic development and population (23,753,700 in 2021) in Jilin Province are at moderate levels when compared to nationwide [21], whereas its mean value for public medical and health efficiency from 2005 to 2017 was among the lowest levels [20].

### Participants

Multistage stratified random cluster sampling procedures were used in each survey. As the primary sampling units for provincial representativeness, the 2008 and 2018 surveys sampled 13 and 12 counties, respectively. In each county, five townships (in rural areas) or streets (in urban areas) were randomly selected. Then, within each township / street, two villages or communities were randomly selected. Finally, within each community or village, 30–40 households in 2008 and 60 households in 2018 were randomly sampled. All members in the selected households were interviewed. A total of 13,922 individuals from 4,778 households participated in the 2008 survey and 17,784 individuals from 7,200 households participated in the 2018 survey. The response rates among participants aged above 15 years were 72.4% and 89.2% in the 2008 and 2018 surveys, respectively.

Women of reproductive age (n = 3,064 in 2008 and n = 6,123 in 2018) were surveyed for their birth history and livebirths within the past five years. Children younger than five years were also surveyed (n = 627 in 2008 and n = 784 in 2018) to collect information on maternal breastfeeding practices and perinatal characteristics, with questions only raised for the last child born in the past five years. The children’s survey was administered by the mother of the child. Children whose mothers were absent from the survey owing to internal migration were further excluded (n = 137 in 2008 and n = 293 in 2018). Finally, there were 490 and 491 mother-child pairs included in the 2008 and 2018 surveys, respectively.

### Variables

The outcome variables included early initiation of breastfeeding, exclusive breastfeeding within the first six months, median duration of breastfeeding, continued breastfeeding at one year, and timely introduction of complementary foods. The definition of each indicator is detailed in Supplementary Table 1. Two indicators (i.e., timing of breastfeeding initiation and early initiation of breastfeeding) were comparable across ten years because both surveys asked the same related questions. However, the remaining outcomes were not comparable between the two surveys. In addition, the duration of breastfeeding could only be calculated in the 2018 survey because there were no relevant questions in 2008. We therefore analysed the determinants separately.

Maternal, children’s and health services’ characteristics of the participants were collected. Maternal variables included age, ethnicity, region, education, occupation, household wealth, self-reported health, and parity. Children’s information included age, gender, and birth weight (low birth weight: 0–2,499 g, normal birth weight: 2,500–3,999 g, macrosomia: >= 4,000 g). Health service indicators included antenatal visits, place of delivery, mode of delivery, and postpartum visits.

### Data analysis

Descriptive statistics were used to describe the characteristics of the participants and breastfeeding practices in 2008 and 2018, respectively. Chi-square tests were applied to assess the differences in the rates of early initiation of breastfeeding and timing of breastfeeding initiation between the two surveys. The median duration of breastfeeding in the 2018 survey was calculated and then transformed into a dichotomous variable using the median (12 months) as a cut-off.

Univariate analyses were performed to assess the differences in breastfeeding practices across maternal, children’s and health services’ characteristics. Crude odds ratios (ORs) and 95% confidence intervals (CIs) were estimated. Multivariate logistic regression was further conducted to explore factors associated with each breastfeeding outcome after controlling for potential confounders. The behaviour of ‘early initiation of breastfeeding’ was also adjusted in the regression models that explored the determinants of later breastfeeding practices, including (a) exclusive breastfeeding within six months; (b) duration of breastfeeding; (c) continued breastfeeding at one year; and (d) timely introduction of complementary foods. Adjusted ORs and 95% CIs were reported in each logistic regression model. All regression analyses were performed separately with the 2008 and 2018 data. A *P*-value of less than 0.05 was considered statistically significant. Data analyses were performed in Stata 13.1.

## Results

### Sample characteristics

Table 1 shows the maternal, children’s and health services’ characteristics of the participants. In both the 2008 and 2018 surveys, the majority of the mothers were 26 years old and above, of Han ethnicity, employed, and primiparous; most children were 24 to 60 months old, male, and of normal birth weight. Mothers in the 2018 survey were more likely to be older, live in urban areas, have a higher education level, be multiparous, perceive their health as poorest / middle, have a higher frequency of antenatal / postpartum clinic visits, and deliver in county- or higher-level hospitals than mothers in the 2008 survey (*P* < 0.05).

**Table 1:** Characteristics of the mother-infant pairs in the National Health Service Surveys (NHSS) conducted in 2008 (n = 490) and 2018 (n = 491) in Jilin Province, China

Characteristics	2018 N (%)	2008 N (%)	*χ2*	*P*-values
**Health services’ characteristics**				
**Antenatal visits**			160.94	**< 0.001**
0–4	70 (14.26)	194 (39.59)		
5–8	183 (37.27)	230 (46.94)		
9-	238 (48.47)	66 (13.47)		
**Place of delivery**			43.79	**< 0.001**
County- or higher-level hospital	370 (75.36)	249 (54.97)		
Maternal and Child Health Hospital	80 (16.29)	142 (31.35)		
Primary hospital / private hospital / home, clinic or other	41 (8.35)	62 (13.69)		
**Mode of delivery**			0.98	0.322
Vaginal	223 (45.42)	236 (48.36)		
Caesarean section	268 (54.58)	252 (51.64)		
**Postpartum visits**			93.25	**< 0.001**
0	107 (21.79)	226 (46.12)		
1–2	250 (50.92)	115 (23.47)		
3	134 (27.29)	149 (30.41)		
**Mothers’ characteristics**				
**Age, years**			68.28	**< 0.001**
15 to < 21	6 (1.22)	5 (1.02)		
21 to < 26	50 (10.18)	148 (30.20)		
26 to < 31	168 (34.22)	163 (33.27)		
≥ 31	267 (54.38)	174 (35.51)		
**Ethnicity**			0.12	0.732
Han Chinese	450 (91.65)	452 (92.24)		
Minority	41 (8.35)	38 (7.76)		
**Region**			20.08	**< 0.001**
Urban	248 (50.51)	178 (36.33)		
Rural	243 (49.49)	312 (63.67)		
**Maternal education**				
Junior school or below	248 (50.51)	348 (71.17)	58.23	**< 0.001**
Senior high school	91 (18.53)	82 (16.77)		
Bachelor degree or above	152 (30.96)	59 (12.07)		
**Occupation**			0.88	0.348
Employed	357 (72.71)	343 (70.00)		
Unemployed	134 (27.29)	147 (30.00)		
**Household wealth** ^**†**^			5.25	0.073
Poorest	194 (39.51)	166 (33.88)		
Middle	164 (33.40)	161 (32.86)		
Richest	133 (27.09)	163 (33.27)		
**Self-reported health** ^**‡**^				
Poorest	22 (4.48)	6 (1.23)	15.52	**< 0.001**
Middle	59 (12.02)	37 (7.60)		
Best	410 (83.50)	444 (91.17)		
**Parity**			26.80	**< 0.001**
Primiparous	266 (54.18)	344 (70.20)		
Multiparous	225 (45.82)	146 (29.80)		
**Children’s characteristics**				
**Age, months**			11.77	0.067
0 to < 6	48 (9.78)	47 (9.67)		
6 to < 12	51 (10.39)	74 (15.23)		
12 to < 24	97 (19.76)	102 (20.99)		
24 to ≤ 60	295 (60.08)	263 (54.12)		
**Gender**			2.40	0.121
Male	261 (53.16)	284 (58.08)		
Female	230 (46.84)	205 (41.92)		
**Birth weight**, %			1.13	0.569
Low birth weight (< 2500 g)	17 (3.46)	12 (2.48)		
Normal birth weight (2,500–3,999 g)	410 (83.50)	414 (85.54)		
Macrosomia (≥ 4000 g)	64 (13.03)	58 (11.98)		

^†^Household wealth: categorised according to annual family income per capita; in 2008, poor: 0–2,833 RMB, middle: 2,833–5,400 RMB, rich: ≥ 5,400 RMB; in 2018, poor: 0–10,000 RMB, middle: 10,000–20,000 RMB, rich: ≥ 20,000 RMB.

^‡^Self-reported health: categorised according to self-reported score; poor: 0–59, middle: 60–79, good: 80–100

### Breastfeeding practices

Breastfeeding practices are illustrated in Table 2. The prevalence of early initiation of breastfeeding was similar between 2008 (27.6%) and 2018 (26.1%) (*P* = 0.895). A smaller proportion of women who initiated breastfeeding within 30 min after birth was found in 2018 (17.2%) than in 2008 (21.9%) (*P* = 0.009). In 2008, according to the WHO definition, the rate of exclusive breastfeeding within six months was only 29.8%, and the rates of continued breastfeeding at one year and timely introduction of complementary foods were 67.5% and 51.4%, respectively. In 2018, 47.6% of children (aged 6–60 months) had been breastfed exclusively within six months. A total of 54.6% of children (aged 12–60 months) continued breastfeeding at one year, and 56.5% (aged 8–60 months) were introduced to complementary foods at six months. The median duration of breastfeeding among children who had stopped being breastfed at the time of the survey was 12 months.

**Table 2:** Breastfeeding practices in 2008 and 2018 in Jilin Province, China

Breastfeeding practices ^a^	2018, %^b^	2008, %^b^	χ2	*P*-values
**Timing of breastfeeding initiation** ^**c**^			13.56	0.009
Within 30 min after birth	17.24	21.93		
Above 30 min to 1 h after birth	8.87	5.70		
Above 1 to 24 h after birth	33.00	27.63		
The second day after birth and later	33.00	37.72		
Never	7.88	7.02		
**Early initiation of breastfeeding** ^**c**^	26.11	27.63	0.02	0.895
**Exclusive breastfeeding within the first six months of life**	47.63	29.79	—	—
**Median duration of breastfeeding, months** ^**d**^	12 (6–16)^e^	—	—	—
**Continued breastfeeding at one year**	54.59	67.50		
**Timely introduction of complementary food**	56.47	51.43	—	—

Abbreviations: *IQR* interquartile range

^a^ Definitions of the breastfeeding practices are fully described in Supplementary Table 1,

^b^ Figures in this column are all percentages unless otherwise stated,

^c^ Variables in the 2008 and 2018 surveys are comparable, as they have the same definition

^d^ Data are presented as the median (IQR). This variable is not available in the 2008 survey,

^e^ IQR, interquartile range (Q_L_, Q_U_)

### Determinants of breastfeeding practices

The determinants of breastfeeding are shown in Tables 3, 4 and 5. After adjusting for potential confounders, early initiation of breastfeeding was positively associated with giving birth in the Maternal and Child Health Hospital (Adjusted OR 3.65; 95% CI 1.51, 8.45) and negatively associated with Caesarean section (Adjusted OR 0.46; 95% CI 0.22, 0.96) in 2018. Early initiation of breastfeeding was positively associated with mothers living in urban regions (Adjusted OR 5.64; 95% CI 1.22, 26.14) in 2008 (Table 3).

**Table 3:** Determinants of early initiation of breastfeeding in 2008 and 2018 in Jilin Province, China^†^

	2018				2008		
	No. (%) Early initiation of breastfeeding	Crude OR (95% CI)	Adjusted OR (95% CI)		No. (%) Early initiation of breastfeeding	Crude OR (95% CI)	Adjusted OR (95% CI)
**Health service characteristics**							
**Antenatal visits**							
0–4	6 (37.50)	1	1		22 (30.56)	1	1
5–8	18 (26.09)	0.59 (0.19, 1.85)	0.75 (0.21, 2.68)		34 (29.06)	0.93 (0.49, 1.77)	0.90 (0.42, 1.93)
9-	29 (24.58)	0.54 (0.18, 1.62)	0.83 (0.23, 2.94)		7 (17.95)	0.50 (0.19, 1.30)	1.15 (0.34, 3.87)
**Mode of delivery**							
Vaginal	36 (35.64)	1	1		31 (33.70)	1	1
Caesarean section	17 (16.67)	0.36 (0.19, 0.70)	**0.46 (0.22, 0.96)**		32 (23.53)	0.61 (0.34, 1.09)	0.78 (0.37,1.62)
**Place of delivery**							
County- or higher-level hospital	35 (22.15)	1	1		29 (24.37)	1	1
Maternal and Child Health Hospital	15 (46.88)	3.10 (1.41, 6.83)	**3.65 (1.51, 8.45)**		16 (23.19)	0.94 (0.47, 1.88)	0.91 (0.42, 1.98)
Primary hospital / private hospital / home, clinic or other	3 (23.08)	1.05 (0.28, 4.04)	0.78 (0.18, 3.34)		9 (39.13)	2.00 (0.78, 5.09)	1.31 (0.46, 3.72)
**Mothers’ characteristics**							
**Region**							
Rural	24 (21.82)	1	1		11 (11.83)	1	1
Urban	29 (31.18)	1.62 (0.86, 3.05)	1.80 (0.72, 4.48)		52 (38.52)	4.67 (2.28, 9.58)	**5.64 (1.22, 26.14)**
**Maternal education**							
Junior school or below	25 (27.17)	1	1		52 (33.33)	1	1
Senior high school	9 (21.43)	0.73 (0.31, 1.74)	0.85 (0.31, 2.30)		7 (17.95)	0.44 (0.18, 1.06)	2.62 (0.56, 16.32)
Bachelor degree or above	19 (27.54)	1.02 (0.51, 2.05)	2.03 (0.73, 5.61)		4 (12.12)	0.28 (0.09, 0.83)	1.68 (0.30, 9.47)

Abbreviations: *CI* Confidence Interval, *OR* Odds Ratio

^†^Variables controlled in the regression models as covariates: maternal age, ethnicity, occupation, household wealth, self-reported health, parity, child’s sex and birth weight

**Table 4:** Determinants of exclusive breastfeeding within the first six months in 2008 and 2018 in Jilin Province, China^†^

	2018				2008		
	No. (%) breastfed exclusively within six months	Crude OR (95% CI)	Adjusted OR (95% CI)		No. (%) breastfed exclusively within six months	Crude OR (95% CI)	Adjusted OR (95% CI)
**Health services’ characteristics**							
**Antenatal visits**							
0–4	40 (60.61)	1	1		4 (36.36)	1	1
5–8	86 (50.89)	0.67 (0.38, 1.20)	0.70 (0.38, 1.29)		8 (27.59)	0.67 (0.15, 2.91)	0.88 (0.05, 15.53)
9-	85 (40.87)	0.45 (0.26, 0.79)	0.55 (0.29, 1.03)		2 (28.57)	0.70 (0.09, 5.43)	8.51 (0.03, 29.41)
**Mode of delivery**							
Vaginal	111 (54.68)	1	1		3 (25.00)	1	1
Caesarean section	100 (41.67)	0.59 (0.41, 0.86)	**0.65 (0.43, 0.98)**		11 (31.43)	1.38 (0.31, 6.09)	0.96 (0.03, 29.04)
**Place of delivery**							
County- or higher-level hospital	161 (47.63)	1	1		9 (45.00)	1	1
Maternal and Child Health Hospital	30 (44.78)	0.89 (0.53, 1.51)	0.78 (0.44, 1.40)		3 (16.67)	0.24 (0.05, 1.12)	0.13 (0.00, 4.93)
Primary hospital / private hospital / home, clinic or other	20 (52.63)	1.22 (0.62, 2.39)	1.31 (0.64, 2.68)		2 (33.33)	0.61 (0.09, 4.14)	0.26 (0.01, 10.93)
**Early initiation of breastfeeding**							
No	131 (40.81)	1	1		11 (30.56)	1	1
Yes	80 (65.57)	2.76 (1.79, 4.27)	**2.65 (1.65, 4.26)**		3 (27.27)	0.85 (0.19, 3.84)	1.07 (0.05, 21.70)
**Postpartum visits**							
0-	53 (51.46)	1	1		8 (36.36)	1	1
1–2	102 (45.13)	0.78 (0.49, 1.24)	0.87 (0.53, 1.45)		4 (30.77)	0.78 (0.18, 3.36)	0.38 (0.01, 13.18)
3-	56 (49.12)	0.91 (0.53, 1.55)	0.84 (0.47, 1.53)		2 (16.67)	0.35 (0.06, 2.01)	0.19 (0.01 ,7.07)
**Mothers’ characteristics**							
**Region**							
Rural	91 (42.13)	1	1		4(25.00)	1	1
Urban	120 (52.86)	1.54 (1.06, 2.24)	1.04 (0.62, 1.75)		10 (32.26)	1.43 (0.37, 5.56)	1.01 (0.00, 210.63)
**Maternal education**							
Junior school or below	119 (52.19)	1	1		11 (31.43)	1	1
Senior high school	34 (43.04)	0.69 (0.41, 1.16)	0.89 (0.50, 1.58)		2 (25.00)	0.73 (0.13, 4.19)	0.32 (0.00, 64.41)
Bachelor degree or above	58 (42.65)	0.68 (0.44, 1.04)	0.84 (0.48, 1.47)		1 (25.00)	0.73 (0.07, 7.80)	0.24 (0.00, 110.48)

Abbreviations: *CI* Confidence Interval, *OR* Odds Ratio

^†^Variables controlled in the regression models as covariates: maternal age, ethnicity, occupation, household wealth, self-reported health, parity, child’s sex and birth weight

**Table 5:** Determinants of a longer duration of breastfeeding^#^, continued breastfeeding at one year^‡^, and timely introduction of complementary foods in 2018 in Jilin Province, China^†^^

	A longer duration of breastfeeding#			Continued breastfeeding at one year^‡^			Timely introduction of complementary foods^‡^	
	Mean duration (SD), months	Adjusted OR (95% CI)		No. (%) Continued breastfeeding at one year	Adjusted OR (95% CI)		No. (%) Timely introduction of complementary foods	Adjusted OR (95% CI)
**Health services’ characteristics**								
**Antenatal visits**								
0–4	63 (56.25)	1		35 (54.69)	1		31 (46.97)	1
5–8	74 (54.01)	0.80 (0.40, 1.58)		81 (55.10)	1.09 (0.58, 2.04)		89 (59.94)	1.27 (0.70, 2.32)
9-	67 (60.36)	0.86 (0.43, 1.74)		98 (54.14)	1.40 (0.73, 2.69)		120 (60.91)	1.44 (0.77, 2.68)
**Mode of delivery**								
Vaginal	100 (59.52)	1		105 (58.99)	1		108 (55.96)	1
Caesarean section	104 (54.17)	0.77 (0.49, 1.22)		109 (50.93)	0.70 (0.45, 1.09)		132 (56.90)	1.08 (0.71, 1.63)
**Place of delivery**								
County- or higher-level hospital	160 (57.97)	1		167 (56.23)	1		186 (57.23)	1
Maternal and Child Health Hospital	25 (46.30)	0.62 (0.33, 1.16)		28 (43.75)	0.57 (0.31, 1.02)		42 (64.62)	1.27 (0.71, 2.29)
Primary hospital / private hospital / home, clinic or other	19 (63.33)	1.05 (0.47, 2.37)		19 (61.29)	1.14 (0.51, 2.52)		12 (34.29)	**0.42 (0.20, 0.89)**
**Early initiation of breastfeeding**								
No	137 (55.02)	1		146 (51.77)	1		1	1
Yes	67 (60.36)	1.32 (0.80, 2.19)		68 (61.82)	1.57 (0.96, 2.57)		1.39 (0.86, 2.23)	1.39 (0.86, 2.23)
**Postpartum visits**								
0-	50 (57.47)	1		50 (54.95)	1		1	1
1–2	96 (53.93)	1.00 (0.57, 1.75)		104(51.49)	0.96 (0.56, 1.65)		0.95 (0.58, 1.58)	0.95 (0.58, 1.58)
3-	58 (61.05)	1.10 (0.58, 2.11)		60 (60.61)	1.09 (0.58, 2.05)		1.39 (0.77, 2.51)	1.39 (0.77, 2.51)
**Mothers’ characteristics**								
**Region**								
Rural	83 (48.54)	1		89 (45.64)	1		126 (61.17)	1
Urban	121 (64.02)	1.63 (0.92, 2.88)		125 (63.45)	**1.92 (1.12, 3.31)**		114 (52.05)	0.77 (0.46, 1.29)
**Maternal education**								
Junior school or below	121 (62.37)	1		126 (62.07)	1		119 (54.09)	1
Senior high school	29 (47.54)	0.68 (0.36, 1.28)		30 (43.48)	0.56 (0.30, 1.05)		43 (57.33)	0.92 (0.51, 1.66)
Bachelor degree or above	54 (51.43)	0.79 (0.43, 1.46)		58 (48.33)	0.73 (0.40, 1.32)		78 (60.00)	0.93 (0.53, 1.65)

Abbreviations: *CI* Confidence Interval, *OR* Odds Ratio, *SD* Standard Deviation

^†^Variables controlled in the regression models as covariates: maternal age, ethnicity, occupation, household wealth, self-reported health, parity, child’s sex and birth weight

^See Supplementary Tables 2–4 for univariate analysis results

^#^No relevant data were collected in the 2008 survey

^‡^Sample size was too small to perform logistic regression in the 2008 survey

Exclusively breastfeeding within the first six months was negatively associated with Caesarean section (Adjusted OR 0.65; 95% CI 0.43, 0.98) and positively associated with early initiation of breastfeeding (Adjusted OR 2.65; 95% CI 1.65, 4.26) in 2018 but not associated with any factors in 2008 (Table 4). A longer duration of breastfeeding was associated with mothers living in urban regions in 2018 in the univariate analyses (Supplementary Table 2), while there was no evidence of significance after adjusting for potential confounders (Table 5). However, continued breastfeeding at one year was independently positively associated with living in urban regions (Adjusted OR 1.92; 95% CI 1.12, 3.31) in 2018 (Table 5). Timely introduction of complementary foods was negatively associated with mothers’ delivery in primary hospital / private hospital / home, clinic or other (Adjusted OR 0.42, 95% CI: 0.20, 0.89) in 2018 (Table 5).

## Discussion

This study demonstrated that breastfeeding practices in Northeast China were below the global average level [4, 5]. Determinants of breastfeeding varied between 2008 and 2018. In 2008, mothers’ urban residence was positively associated with early initiation of breastfeeding. In 2018, breastfeeding determinants included more factors, including urban residence, mode of delivery, and place of delivery, where early initiation of breastfeeding took the most important role.

In the present study, the rates of exclusive breastfeeding during the first six months in 2008 and 2018 were below the WHO’s target of 50% towards 2025 [22], and the prevalence of early initiation of breastfeeding was among the ‘poor’ levels according to the WHO standard (poor: <= 29%, medium: 30–49%, good: 50–89%, excellent: >= 90%) [23]. In comparison with the Chinese national data of U5CNHSS (34.9%) [8] and regional data of Nanning (37%) [11] in 2018, exclusive breastfeeding in Northeast China in 2018 (47.6%) was higher. Our result was consistent with the U5CNHSS finding that the prevalence of exclusive breastfeeding was higher in the east (39.3%) than in the west (25.7%) [8]. In addition, the rates of early initiation of breastfeeding in our study (< 28%) were lower than the U5CNHSS in 2013–18 [8] but higher than the population-based survey across 12 regions in 2017–18 [6]. Importantly, the present study found a significant decrease in breastfeeding initiation within 30 min from 2008 to 2018. Therefore, promoting the practice of early skin-to-skin contact, early suckling, and early initiation of breastfeeding is urgently needed in Northeast China.

This study highlighted the variation in breastfeeding determinants between 2008 and 2018. Breastfeeding was mainly influenced by socioeconomic factors (i.e., region of residence) in 2008, while additional factors related to health system determinants (i.e., early initiation of breastfeeding, delivering in the Maternal and Child Health Hospital, and mode of delivery) were found in 2018. The changed role of health system factors suggested that public health services are an important area for breastfeeding promotion in Northeast China in the context of health system reform. Importantly, the key role of early initiation of breastfeeding revealed in our 2018 survey [6, 7], consistent with the population-based survey in 2018, showed that early initiation of breastfeeding was a major determinant of exclusive breastfeeding within the first six months. We found that early initiation of breastfeeding was positively associated with giving birth in the Maternal and Child Health Hospital (Adjusted OR = 3.65) but negatively associated with caesarean section (Adjusted OR = 0.46) in 2018. Since 2010 [24], the Chinese government has been putting forward comprehensive measures, including promoting postpartum breastfeeding guidance, which target birth delivery hospitals to control rising caesarean sections, with Maternal and Child Health Hospitals acting as the main control knob. The decreasing caesarean rates in Jilin indicates these efforts suggest that measures targeting health facilities seem to be associated with better breastfeeding practice. Nevertheless, results for breastfeeding initiation within 30 min and one hour were still reported as being low in our 2018 survey, suggesting that breastfeeding interventions that apply the baby-friendly hospitals’ strategies warrant further consideration. By 2015, a total of 21 medical institutions in Jilin Province had been entitled baby-friendly hospitals [25]. Increasing the number of baby-friendly hospitals and continued implementation of the BFHI policy in maternity hospitals in Jilin Province are urgently needed.

We found no evidence of association between antenatal / postpartum visits and breastfeeding practices in the 2008 and 2018 surveys. Neither population-based [6, 7, 10, 26] nor regional [11] surveys in China in 2018 revealed an impact of postpartum visits on breastfeeding. Even though the effectiveness of antenatal and postnatal care in increasing breastfeeding rates has been recognised, according to the most recent Cochrane reviews, the consistency of evidence is low [27], and breastfeeding support is more likely to be effective among mothers with high initiation rates [28, 29]. These findings suggest that the community-based approach might not be sufficient to promote breastfeeding in China’s current context. Owing to the emerging evidence on the effectiveness of BFHI policy in increasing breastfeeding rates in China [30, 31], our data support the belief that a combination of BFHI and community health care approaches can enhance breastfeeding in China to a greater extent. This augmentation is also supported by a growing number of trials using a multisectoral approach in low- and middle-income settings [32, 33].

This study has some strengths. First, our results were derived from two provincially representative survey datasets with a standardised sampling method. Second, comparison of some outcomes across two surveys could reveal the differences in breastfeeding practices over the most recent ten years. Third, the 2018 survey results provided the latest information on breastfeeding practices in Jilin Province in China and evidence for the importance of early initiation of breastfeeding in achieving optimal breastfeeding practices.

Limitations of this study should be acknowledged. Due to the differences in questions in the 2008 and 2018 surveys, some breastfeeding indicators were not comparable between the two years, and no comparison analysis was performed. In addition, logistic regression analyses were unable to be performed for some variables in the 2008 survey owing to the small sample size. Finally, some breastfeeding indicators in 2018 were not calculated according to the WHO definition, and the generalisability and comparability of these results were thus restricted.

## Conclusion

In conclusion, breastfeeding practices in Northeast China are below the world average levels. The rate of breastfeeding initiated within 30 min decreased significantly from 2008 to 2018. Our study adds to the literature that early initiation of breastfeeding is beneficial to the practices of exclusive breastfeeding within six months. The Chinese government has been focused on the community-based approach to promote optimal breastfeeding in the last decade. Our data, however, suggest that an institution-based approach should not be substituted by the community-based one in the formulation of breastfeeding strategies in China.

## Electronic supplementary material

Below is the link to the electronic supplementary material.

Supplementary Material 1

## Data Availability

The datasets used and / or analysed during the current study are available from the corresponding author on reasonable request.

## List of Abbreviations

BFHI: Baby-friendly hospital initiative
NHSS: China’s National Health Services Surveys
CI: Confidence Interval
OR: Odds Ratio
SD: Standard deviation
U5CNHSS: Under-5 Child Nutrition and Health Surveillance System
UNICEF: United Nations Children’s Fund
WHO: World Health Organization
