# Supplementary material for: Breastfeeding practices in Northeast China in 2008 and 2018: cross-sectional surveys to explore determinants over a decade
Source: Int Breastfeed J. 2023 May 2;18:25. doi: 10.1186/s13006-023-00562-4 (PMC10155397; doi:10.1186/s13006-023-00562-4)
Supplement: Supplementary file 1 — Supplementary Material 1 [file 13006_2023_562_MOESM1_ESM.docx]

**Supplementary Materials**

**Supplementary Table 1. Definitions of breastfeeding practices.**

| **Indicators** | **Definitions** | |
| --- | --- | --- |
|  | **2008 survey** | **2018 survey** |
| **Timing of breastfeeding initiation ^a, b^** | Timing of breastfeeding initiation of children who were ever breastfed amongst children born in the last 24 months | Timing of breastfeeding initiation of children who were ever breastfed amongst children born in the last 24 months |
| **Early initiation of breastfeeding ^a, c^** | Proportion of children born in the last 24 months who were put to the breast within one hour after birth | Proportion of children born in the last 24 months who were put to the breast within one hour after birth |
| **Exclusive breastfeeding within the first six months of life ^d^** | Proportion of infants 0-5 months of age who were fed exclusively with breast milk ^c^ | Proportion of infants 6-60 months of age who have been fed exclusively with breast milk within the first 6 months of life |
| **Continued breastfeeding at one year ^d^** | Proportion of children 12-15 months of age who were fed with breast milk ^c^ | Proportion of children 12-60 months of age who were fed with breast milk |
| **Median duration of breastfeeding, months ^e^** | — | Median duration of breastfeeding among children who have stop being breastfed |
| **Timely introduction of complementary foods ^d^** | Proportion of infants 6-8 months of age who receive solid, semi-solid or soft foods during previous day ^c^ | Proportion of infants 8-60 months of age who started receiving solid, semi-solid or soft foods from 6-8 months of age |

^a^ Definitions of this indicator are the same in the 2008 and 2018 surveys.

^b^ Self-defined by the authors of this paper.

^c^ In line with the WHO’s definition.

^d^ Definitions of this indicator are different in the 2008 and 2018 surveys.

^e^ Data were not available in the 2008 survey.

**Supplementary Table 2. Factors associated with a longer duration of breastfeeding in 2018 in Jilin Province, China, univariate analysis.**

|  | **Mean duration (SD), months** | **Crude OR (95%CI)** |
| --- | --- | --- |
| **Health services' characteristics** |  |  |
| **Antenatal visits** |  |  |
| 0-4 | 63(56.25) | 1 |
| 5-8 | 74(54.01) | 0.72(0.37,1.39) |
| 9- | 67(60.36) | 0.63(0.34,1.19) |
| **Mode of delivery** |  |  |
| Vaginal | 100(59.52) | 1 |
| Caesarean section | 104(54.17) | 0.80(0.53,1.22) |
| **Place of delivery** |  |  |
| County- or higher-level hospital | 160(57.97) | 1 |
| Maternal and Child Health Hospital | 25(46.30) | 0.63(0.35,1.12) |
| Primary hospital/Private hospital/Home, clinic or other | 19(63.33) | 1.25(0.57,2.73) |
| **Early initiation of breastfeeding** |  |  |
| No | 137(55.02) | 1 |
| Yes | 67(60.36) | 1.24(0.79,1.96) |
| **Postpartum visits** |  |  |
| 0- | 50(57.47) | 1 |
| 1-2 | 96(53.93) | 0.87(0.52,1.45) |
| 3- | 58(61.05) | 1.16(0.64,2.10) |
| **Mothers' characteristics** |  |  |
| **Region** |  |  |
| Rural | 83(48.54) | 1 |
| Urban | 121(64.02) | **1.89(1.24,2.88)** |
| **Maternal education** |  |  |
| Junior school or below | 121(62.37) | 1 |
| Senior high school | 29(47.54) | 0.55(0.31,0.98) |
| Bachelor degree or above | 54(51.43) | 0.64(0.40,1.03) |

**Supplementary Table 3. Factors associated with continued breastfeeding at one year in 2008 and 2018 in Jilin Province, China, univariate analysis.**

|  | **2018** | |  | **2008** | |
| --- | --- | --- | --- | --- | --- |
|  | **No.(%)Continued breastfeeding at 1 year** | **Crude OR (95%CI)** |  | **No.(%)Continued breastfeeding at 1 year** | **Crude OR (95%CI)** |
| **Health services' characteristics** |  |  |  |  |  |
| **Antenatal visits** |  |  |  |  |  |
| 0-4 | 35(54.69) | 1 |  | 11(73.33) | 1 |
| 5-8 | 81(55.10) | 1.02(0.56,1.83) |  | 14(73.68) | 1.02(0.22,4.72) |
| 9-12 | 98(54.14) | 0.98(0.55,1.73) |  | 2(33.33) | 0.18(0.02,1.41) |
| **Mode of delivery** |  |  |  |  |  |
| Vaginal | 105(58.99) | 1 |  | 15(68.18) | 1 |
| Caesarean section | 109(50.93) | 0.72(0.48,1.08) |  | 12(66.67) | 0.93(0.25,3.52) |
| **Place of delivery** |  |  |  |  |  |
| County- or higher-level hospital | 167(56.23) | 1 |  | 13(61.90) | 1 |
| Maternal and Child Health Hospital | 28(43.75) | 0.61(0.35,1.04) |  | 8(80.00) | 2.46(0.41,14.63) |
| Primary hospital/Private hospital/Home, clinic or other | 19(61.29) | 1.23(0.58,2.63) |  | 4(66.67) | 1.23(0.18,8.33) |
| **Early initiation of breastfeeding** |  |  |  |  |  |
| No | 146(51.77) | 1 |  | 17(60.71) | 1 |
| Yes | 68(61.82) | 1.51(0.96,2.37) |  | 10(83.33) |  |
| **Postpartum visits** |  |  |  |  |  |
| 0- | 50(54.95) | 1 |  | 6(37.50) | 1 |
| 1-2 | 104(51.49) | 0.87(0.53,1.43) |  | 9(81.82) | 7.50(1.20,47.05) |
| 3- | 60(60.61) | 1.26(0.71,2.25) |  | 12(92.31) | 20.00(2.05,195.00) |
| **Mothers' characteristics** |  |  |  |  |  |
| **Region** |  |  |  | 8(47.06) | 1 |
| Rural | 89(45.64) | 1 |  | 19(82.61) | 5.34(1.27,22.52) |
| Urban | 125(63.45) | 2.07(1.38,3.10) |  |  |  |
| **Maternal education** |  |  |  |  |  |
| Junior school or below | 126(62.07) | 1 |  | 21(70.00) | 1 |
| Senior high school | 30(43.48) | 0.47(0.27,0.82) |  | 3(50.00) | 0.43(0.07,2.54) |
| Bachelor degree or above | 58(48.33) | 0.57(0.36,0.90) |  | 3(75.00) | 1.29(0.12,14.09) |

**Supplementary Table 4. Factors associated with timely introduction of complementary foods in 2008 and 2018 in Jilin Province, China, univariate analysis**

|  | **2018** | |  | **2008** | |
| --- | --- | --- | --- | --- | --- |
|  | **No.(%)Timely introduction of complementary foods** | **Crude OR (95%CI)** |  | **No.(%)Timely introduction of complementary foods** | **Crude OR (95%CI)** |
| **Health services' characteristics** |  |  |  |  |  |
| **Antenatal visits** |  |  |  |  |  |
| 0-4 | 31(46.97) | 1 |  | 4(36.36) | 1 |
| 5-8 | 89(59.94) | 1.38(0.78,2.44) |  | 13(65.00) | 3.25(0.70,15.07) |
| 9-12 | 120(60.91) | 1.76(1.00,3.09) |  | 1(25.00) | 0.58(0.04,7.66) |
| **Mode of delivery** |  |  |  |  |  |
| Vaginal | 108(55.96) | 1 |  | 7(43.75) | 1 |
| Caesarean section | 132(56.90) | 1.04(0.71,1.53) |  | 11(57.89) | 1.77(0.46,6.78) |
| **Place of delivery** |  |  |  |  |  |
| County- or higher-level hospital | 186(57.23) | 1 |  | 13(59.09) | 1 |
| Maternal and Child Health Hospital | 42(64.62) | 1.36(0.78,2.37) |  | 1(14.29) | 0.12(0.01,1.13) |
| Primary hospital/Private hospital/Home, clinic or other | 12(34.29) | 0.39(0.19,0.81) |  | 2(66.67) | 1.38(0.11,17.67) |
| **Early initiation of breastfeeding** |  |  |  |  |  |
| No | 167(54.22) | 1 |  | 14(56.00) | 1 |
| Yes | 73(62.39) | 1.40(0.91,2.17) |  | 4(40.00) | 0.52(0.12,2.33) |
| **Postpartum visits** |  |  |  |  |  |
| 0- | 55(54.46) | 1 |  | 6(50.00) | 1 |
| 1-2 | 118(54.88) | 1.02(0.63,1.64) |  | 6(66.67) | 2.00(0.33,11.97) |
| 3- | 67(61.47) | 1.33(0.77,2.31) |  | 6(42.86) | 0.75(0.16,3.53) |
| **Mothers' characteristics** |  |  |  |  |  |
| **Region** |  |  |  |  |  |
| Rural | 126(61.17) | 1 |  | 10(62.50) | 1 |
| Urban | 114(52.05) | 0.69(0.47,1.01) |  | 8(42.11) | 0.44(0.11,1.70) |
| **Maternal education** |  |  |  |  |  |
| Junior school or below | 119(54.09) | 1 |  | 11(50.00) | 1 |
| Senior high school | 43(57.33) | 1.14(0.67,1.94) |  | 2(40.00) | 0.67(0.09,4.80) |
| Bachelor degree or above | 78(60.00) | 1.27(0.82,1.98) |  | 5(62.50) | 1.67(0.32,8.74) |
